# Emergence of Arctic-like Rabies Lineage in India

**DOI:** 10.3201/eid1301.060702

**Published:** 2007-01

**Authors:** Susan A. Nadin-Davis, Geoff Turner, Joel P. V. Paul, Shampur N. Madhusudana, Alexander I. Wandeler

**Affiliations:** * Canadian Food Inspection Agency, Ottawa, Ontario, Canada; † National Institute for Mental Health; Neurosciences, Bangalore, India; 1 Current affiliation: University College London Hospitals NHS Trust, London, United Kingdom

**Keywords:** human and dog rabies, molecular epidemiology, rabies virus Arctic lineage, research

## Abstract

A collection of 37 rabies-infected samples, 10 human saliva and 27 animal brain, were recovered during 2001–2004 from the cities of Bangalore and Hyderabad in southern India and from Kasauli, a mountainous region in Himachal Pradesh, northern India. Phylogenetic analysis of partial N gene nucleotide sequences of these 37 specimens and 1 archival specimen identified 2 groups, divided according to their geographic (north or south) origins. Comparison of selected Indian viruses with representative rabies viruses recovered worldwide showed a close association of all Indian isolates with the circumpolar Arctic rabies lineage distributed throughout northern latitudes of North America and Europe and other viruses recovered from several Asian countries.

## Abstract 2

Progenitors of Arctic-like rabies viruses, which now circulate extensively in India, may have been responsible for the emergence of the Arctic rabies lineage.

An estimated 55,000 people, mostly in Asian countries, die of rabies each year (*1*). The etiologic agent of this disease is rabies virus or a closely related member of the *Lyssavirus* genus; various rabies virus variants, which circulate widely in many dog populations throughout Asia (*2*), are responsible for most human infections. Although rabies is preventable, the high cost of postexposure prophylaxis, compounded by the lack of education and awareness about rabies, limits use of postexposure prophylaxis in many developing countries. Moreover, visitors to these countries are also sometimes unaware of the rabies risk posed by dog bites and thus may not seek appropriate medical attention for such bites. The occasional cases of rabies reported in industrialized countries, such as the United Kingdom, are often the result of exposure while traveling in developing countries such as India (*3**,**4*). In Germany, a recent case of rabies in a person who had visited India remained unidentified until after the patient’s death; soft tissue transplantation from this patient resulted in rabies transmission to several organ recipients (*5*).

Despite the availability of techniques to improve the global rabies situation, limitations in surveillance and epidemiologic investigations impede the institution of such measures (*6*). In industrialized countries, diagnosis of rabies in animals is achieved by using rabies-specific fluorescein-conjugated antibody to detect viral antigen in brain smears; however, antemortem diagnosis in humans must rely on less-invasive methods. The utility of PCR-based methods to detect rabies virus sequences in saliva and other body fluids has been reported (*7*), and PCR is being used in many industrialized countries (*8**,**9*). An additional component of rabies control in such countries is the application of viral typing methods to identify viral variants that circulate in specific host reservoirs (*10*). Knowledge of the association of specific variants with animal hosts has led to increasingly effective control measures that target the hosts responsible for spreading this disease (*11*). Moreover, molecular epidemiologic approaches have enabled study of the spread of certain rabies virus variants and their incursion into new geographic regions (*12*). Adaptation of such methods in developing countries would help provide reliable data on the true extent of rabies in such countries, provide epidemiologic data about the spread of rabies, and justify allocation of increased resources.

Recently, a national rabies survey in India, based on clinical diagnosis and sponsored by the World Health Organization, found that 20,000 persons died of rabies each year (*13*). These observations indicate a great need to strengthen laboratory diagnostic capabilities for rabies in India and to use genetic typing to improve knowledge of the nature of the viruses that circulate in India. The resulting increase in disease surveillance would help justify subsequent control measures. Accordingly, molecular methods for rabies virus detection have been introduced to the National Institute for Mental Health and Neurosciences in Bangalore, India. Using several positive samples identified by this method, we studied the epidemiologic origins of rabies from multiple areas of the country.

## Materials and Methods

### Sample Collection

Rabies was diagnosed by direct fluorescent antibody (DFA) test (*14*) in 27 animal brains (Table) recovered from 2 locations in India: the city of Bangalore (and its surrounding 15 km) and the northern community of Kasauli in the state of Himachal Pradesh. An archival bovine sample from an unknown location in India was included in the study.

**Table:** Rabies samples from India examined in this study

Sample	Date of isolation	Source	Location	GenBank accession no.
Animal brain				
IND01-1	2001	Dog	Bangalore	DQ521215
IND01-18	2001	Dog	Bangalore	DQ521216
IND01-29	2001	Dog	Bangalore	DQ521217
IND01-56	2001	Dog	Bangalore	DQ521218
IND01-63	2001	Dog	Bangalore	DQ521219
IND01-85	2001	Dog	Bangalore	DQ521220
IND01-90	2001	Dog	Bangalore	DQ521221
IND01-91	2001	Dog	Bangalore	DQ521222
IND02-08	2002	Dog	Bangalore	DQ521223
IND02-55	2002	Dog	Bangalore	DQ521224
IND02-62	2002	Dog	Bangalore	DQ521225
IND02-84	2002	Dog	Bangalore	DQ521226
IND02-85	2002	Dog	Bangalore	DQ521227
IND03-08	2003	Dog	Bangalore	DQ521228
IND03-20	2003	Dog	Bangalore	DQ521229
IND03-24	2003	Dog	Bangalore	DQ521230
IND03-37	2003	Dog	Bangalore	DQ521231
IND03-38	2003	Dog	Bangalore	DQ521232
IND04-53	2004	Dog	Bangalore	DQ521233
IND04-57	2004	Dog	Bangalore	DQ521234
IND04-66	2004	Dog	Bangalore	DQ521235
IND04-72	2004	Dog	Bangalore	DQ521236
INDN1	2004	Dog	Kasauli	DQ521237
INDN2	2004	Dog	Kasauli	DQ521238
INDN3	2004	Dog	Kasauli	DQ521239
INDN5	2004	Cat	Kasauli	DQ521240
INDN6	2004	Bovine	Kasauli	DQ521241
V458IND*	1991	Bovine	(Unknown)	AY854599
Human saliva (antemortem)				
INDH10	12 Feb 2004	Female, 40 y	Bangalore	DQ521242
INDH13	20 Feb 2004	Male, 13 y	Hyderabad	DQ521243
INDH14	21 Feb 2004	Male, 30 y	Bangalore	DQ521244
INDH19	17 Mar 2004	Female, 21y	Hyderabad	DQ521245
INDH20	17 Mar 2004	Male, 14 y	Hyderabad	DQ521246
INDH26	3 Apr 2004	Female, 20 y	Hyderabad	DQ521247
INDH27	15 Apr 2004	Male, 8 y	Hyderabad	DQ521248
INDH28	14 Apr 2004	Female, 12 y	Hyderabad	DQ521249
INDH33	25 Apr 2004	Female, 23 y	Hyderabad	DQ521250
INDH34	27 Apr 2004	Male, 27 y	Bangalore	DQ521251

*Archival specimen from unrecorded location.

Antemortem saliva samples were obtained from 37 human patients with clinical signs consistent with a diagnosis of rabies. All patients were located at 1 of 3 hospitals in Bangalore or at 1 hospital in Hyderabad. The molecular methods described below confirmed 10 of these samples (Table) as rabies infected.

### Molecular Characterization of Viruses

Total RNA was recovered from each specimen by using TRIzol reagent (for animal brain tissue) or TRIzol LS reagent (for human saliva samples) as recommended by the supplier (Invitrogen, Burlington, Ontario, Canada). Standard reverse transcription–PCR (RT-PCR) was used to amplify the complete N gene of rabies virus as previously described (*15*). Universal primers RabNfor/RabNrev, shown to be useful for amplification of a wide range of rabies virus strains, were used to perform a nested second round of PCR (*15*). In initial trials, when DFA-positive samples from dogs in Bangalore were used, most samples (17 of 22) required 2 rounds of PCR to generate a visible amplicon; hence, all subsequent analyses routinely incorporated a nested protocol, and samples were scored for presence of rabies only after the second round of PCR. For nucleotide sequencing, 5 μL of the nested PCR product was spotted onto Whatman (Brentford, UK) no.1 filter paper, air-dried, and transferred to the laboratory in Canada. Each PCR product was eluted from the filter paper into 50 μL of RNase-free water and reamplified by using the nested primer set. Final products were purified by using a Wizard PCR Preps Purification System (Promega, Madison, WI, USA). Nucleotide sequencing was performed with an NEN model 4200L automated sequencing system (Li-Cor Biosciences, Lincoln, NE, USA) and IR700/800-labeled primers (Li-Cor Biosciences), based on either the universal primers or the internal N gene sequence, together with a Thermosequenase cycle sequencing kit (Amersham Biosciences, Baie d’Urfé, Quebec, Canada).

Nucleotide sequences were aligned by using CLUSTALX v1.8 (*16*), and phylogenetic analysis was accomplished by using the neighbor-joining algorithm of the PHYLIP 3.61 software package (*17*). Trees were displayed using TREEVIEW (*18*).

## Results

**Figure 1:**
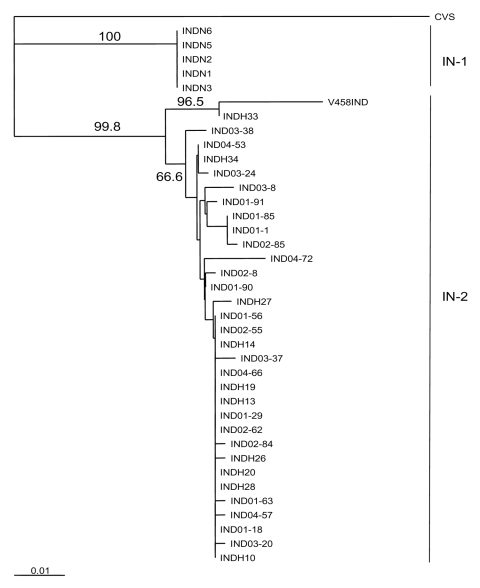
Neighbor-joining tree for 500 bp of nucleoprotein gene sequence for 38 rabies virus samples from India, as described in the Table, using the CVS sequence as an out-group. The sequence window used corresponded to positions 279 to 778 of the CVS reference sequence. Bootstrap values >65% for 1,000 resamplings of the data are shown on branches to the right of the corresponding sample clusters. The 2 main Indian clusters identified by this analysis (IN-1 and IN-2) are indicated to the right of the tree. A genetic distance scale is indicated at bottom left.

All 38 samples (Table), including the archival specimen (V458IND), were confirmed rabies positive by using nested PCR to amplify a portion of the viral N gene. The nucleotide sequence of a 500-base segment in all amplicons was determined for each. These aligned sequences were subjected to phylogenetic analysis using a neighbor-joining algorithm with the CVS strain of rabies included as an out-group. The Indian samples formed 2 main clades (Figure 1). The 5 samples from northern India (IN-1), which were identical over the portion of genome characterized (100% homology), clearly segregated from the main cluster (IN-2) that comprised the more heterogeneous southern isolates. Members of IN-2 exhibited homologies ranging from 94.8% to 100% and showed no segregation according to location of origin. However, 2 specimens, the archival isolate V458IND and a recent human specimen INDH33, were clearly the most distinctive of the group and formed a strongly supported subgroup within this cluster. Intergroup (IN-1 and IN-2) sample homologies ranged between 91.2% and 93.6%.

**Figure 2:**
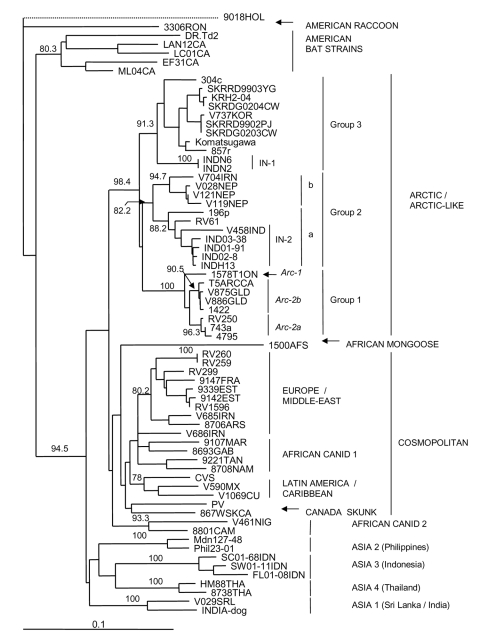
Neighbor-joining tree for 460 bp of nucleoprotein gene sequence for 67 rabies viruses, including representative samples from India, and a European bat lyssavirus type 2 (EBLV-2) specimen, 9018HOL, used as an out-group. The latter branch is shown as a dotted line to indicate that its length has been shortened to permit more detailed illustration of the rest of the tree. All additional rabies viruses used in this analysis are described in the Appendix Table. Bootstrap values >70% for 1,000 resamplings of the data are shown on branches corresponding to the sample clusters. Strains and variants described in the text and in the Appendix Table are illustrated to the right of the tree. A genetic distance scale is indicated at bottom left. Subdivision of the Arctic group 1 into 3 subgroups—Arc-1, Arc-2a, and Arc-2b as described previously (*19*)—is shown in italics.

Because extensive N gene sequence information for rabies viruses is available in publicly accessible databases, similar phylogenetic methods could be used to compare selected Indian isolates with rabies viruses representative of many strains that currently circulate throughout the world (Appendix Table). The tree of Figure 2 was generated by using a shortened sequence window to accommodate variants for which only partially overlapping sequences were available. As shown in Figure 2, all the Indian isolates of this study clustered within a clade designated as Arctic/Arctic-like and were well separated in evolutionary terms from the cosmopolitan lineage as well as other lineages that circulate in various parts of Southeast Asia. One cluster in particular (ASIA1), composed of 2 specimens from dogs of Sri Lanka and Madras (INDIA-Dog), clearly segregated independently of the isolates examined in this study.

Within the Arctic/Arctic-like clade, 3 main groups were strongly supported by bootstrap analysis. Group 1 comprised all North American specimens (from Ontario, northern Canada, and Alaska), specimens from Greenland, and 2 specimens from the former Soviet Union (from Yakutia in the north and Tuva in the south). A prior analysis of Arctic specimens (*19*) strongly supported further division of this group into 3 subgroups designated here as Arc-1, Arc-2a, and Arc-2b. Close association was noted among specimens in subgroup Arc-2a, which originated from Alaska (4795) and the former Soviet Union (RV250 and 743a).

Group 2 comprised specimens from the northeast corner of Iran, where incursion of Arctic-like lineage rabies was recently discovered (*20*); Nepal; Pakistan (sample 196p); and members of the Indian group IN-2 together with 1 additional Indian isolate (RV61) that was recently described (*21*). Support for further subdivision of this group, by which the Indian and Pakistani specimens (subgroup 2a) segregated from the Iranian and Nepalese specimens (subgroup 2b), was strong (bootstrap values of 88.2 and 94.7 for each group, respectively).

Group 3 included the Komatsugawa strain recovered in Japan some years ago; 6 isolates from Korea; and 2 specimens from different regions of the former Soviet Union, Chita (304c), and Chabarovsk (857r). Two members of the northern Indian group (IN-1) of this study formed an outlying branch closely associated with this group.

## Discussion

This study benefited from an initiative to explore the utility of PCR technology for antemortem diagnosis of rabies in human saliva samples. Of 37 suspected rabies cases, 10 were confirmed positive by this technique. Unfortunately, no subsequent follow-up of these patients or postmortem analysis of brain material by DFA was possible. At least some of these patients for whom the saliva test was negative for rabies had likely contracted rabies but had no detectable shedding of virus in saliva during the period of saliva collection. Thus, using these data to infer rabies incidence in humans is difficult. Further application of this method, together with improved follow-up of patient outcome, is needed.

Previous reports (*19**,**21**,**22*) indicated that rabies viruses belonging to the Arctic/Arctic-like lineage are widely dispersed throughout the Northern Hemisphere and are not limited to Arctic regions. Indeed, of the very few genetically characterized isolates originating from India and neighboring countries such as Pakistan (e.g., RV61 and 196p, which are included for comparison in this report), most appeared to be related to the Arctic lineage. However, most of the characterized isolates have been recovered from travelers after their return to developed countries. Ours is the first comprehensive genetic analysis of substantial numbers of isolates directly recovered from several locations in India; our study confirms extensive circulation of the Arctic-like rabies virus lineage in 3 geographically separate areas of India.

**Figure 3:**
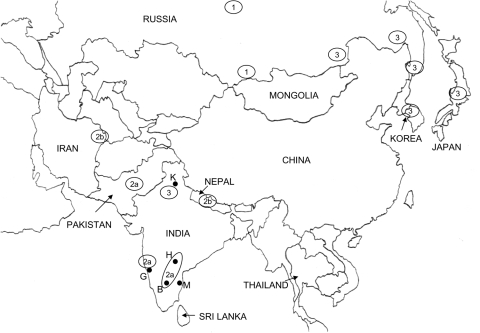
Map of mainland Asia. The locations from which Arctic and Arctic-like variants of the rabies virus have been recovered are shown in circles or ovals with the group designation (1, 2a, 2b, or 3) indicated in the center. Generation of this map was achieved in part by compiling data from 2 previous publications (*19**,**21*). Viral phylogroups previously designated as A and B (*21*) are equivalent to groups 1 and 3, respectively, in this study. B, Bangalore, G, Goa; H, Hyderabad; K, Kasauli; M, Madras.

Phylogenetic analysis identified 3 groups of viruses belonging to the Arctic/Arctic-like rabies virus lineage. A map showing the known distribution of all 3 of these groups throughout Asia is illustrated in Figure 3. This map was compiled from data generated in this study and from previous reports (*4**,**19**–**23*). Because some common specimens were incorporated in many of these analyses, we could surmise the phylogroup membership of many previously described isolates according to the group classification described here (Figure 3).

The viruses that can be considered as the true Arctic strain (group 1) circulate extensively in northern areas of Russia but have also been found in the Tuva region just north of western Mongolia. These viruses are closely related to all the Arctic strain viruses recovered from the Americas. With the exception of the sample from Madras, all Indian isolates recovered from the south of the country, including 1 from a tourist visiting Goa (*4*), belonged to subgroup 2a and were thus closely related to the few characterized viruses recovered from Pakistan. A related but slightly more distant group of viruses (subgroup 2b) was recovered from Nepal and northeastern Iran. Group 3 viruses of the Arctic/Arctic-like lineage circulate extensively in northeastern Asia, Korea (*20**,**23*), and parts of Russia (*21*), and were present in Japan (Komatsugawa isolate) before rabies was eradicated from the country. Perhaps our most surprising finding was that the northern Indian samples were more closely related to these group 3 viruses than to the viruses circulating in southern India and neighboring Nepal. These patterns of viral variant distribution may reflect migrations, recent and historic, and movements of humans and their animals throughout the region. The incursion of 2 separate variants into northern India and Nepal might be a consequence of difficult access between these 2 areas due to the regional terrain. Moreover, it appears likely that group 3 viruses circulate more extensively throughout Asia than is presently documented; further analysis of specimens from the region, especially from China, Mongolia, and Russia, will be needed to form a more complete picture of the spread of this variant throughout the region. A recent study of several Chinese isolates indicated that the circulating virus variants were related to those of Southeast Asia (e.g., ASIA 4) and to the cosmopolitan lineage, but no representatives of the Arctic lineage were found (*24*). Because that study examined rabies viruses recovered only from the southeastern region of China, the possibility remains that the northern regions of this country harbor Arctic-like variants.

The evolutionary mechanisms underlying these phylogenetic patterns can only be speculated upon at this time. Observations made in Canada throughout the 20th century (*12**,**25**,**26*) have documented frequent movement of the Arctic rabies lineage from northern regions to the south, by transmission among populations of red and arctic foxes. Similarly, this lineage could have moved southward from Siberia or other northern latitudes of the former Soviet Union into Nepal, India, and other Asian countries by means of a species jump from the fox to the dog at some point during this spread. However, the tree in Figure 2 provides some argument against this hypothesis. First, within the Arctic/Arctic-like clade, all specimens from temperate and Arctic regions are restricted to group 1 and exhibit more limited genetic variation than that observed for the Asian specimens that are represented in all groups. Although no a priori reason exists to rule out the possibility that rabies can jump from wild-life species to dogs, recent surveillance reports suggest that successful rabies species jumps most often occur from dogs to wildlife (*27**,**28*). Thus, consideration should be given to the possibility that the “Arctic” lineage of rabies first emerged in southern Asia in dogs and that it subsequently spread to northern climes, where it is now maintained by fox populations. The future acquisition of additional data on rabies viruses from Asia should provide the dataset required for a robust molecular clock analysis to explore these hypotheses. Transmission of rabies from a wild fox to a human has been documented in central India (*29*). Given the relatively close phylogeny between rabies virus variants of the Indian dog and arctic fox, further consideration might be given to the role of wildlife in maintaining rabies in India.

A single Indian rabies specimen, INDIA-dog recovered from Madras on the southeastern Indian coast (*30*), clustered with an isolate (V029SRL) typical of a distinct variant found in Sri Lanka (*31*) rather than with the other Indian isolates described in this study. Movement of humans and their animals between Sri Lanka and India, particularly within the southeastern coastal area of the mainland, may have resulted in the movement of this variant between these 2 geographically separate regions. Further studies may show regional circulation of this or other rabies virus variants within India.

We hope that this report will encourage further studies that apply these molecular approaches to the diagnosis of additional rabies cases and the characterization of viruses recovered from other parts of India. Increased knowledge of the complexity of the rabies situation in India should spur efforts to improve public awareness and to better control this disease. Moreover, the data presented here promise to alter current paradigms about the emergence of Arctic rabies.

## Supplementary Material

Appendix Table Additional virus samples from other countries included in the phylogenetic analysis
