# Supplementary material for: Emergence of Arctic-like Rabies Lineage in India
Source: Emerg Infect Dis. 2007 Jan;13(1):111–6. doi: 10.3201/eid1301.060702 (PMC2725804; doi:10.3201/eid1301.060702)
Supplement: Appendix Table — Additional virus samples from other countries included in the phylogenetic analysis [file 06-0702_appT-s1.pdf]

Appendix Table. Additional virus samples from other countries included in the phylogenetic analysis

Grouping	Sample identification	Details (reference no.)	GenBank accession no.
American raccoon	3306RON	Raccoon, Canada, 1999 (1)	AF351826
American bats	DR.Td2	Bovine, Trinidad, 1995 (2)	AF351852
	EF31CA	Big brown bat, Canada, 1989 (2)	AF351831
	LAN12CA	Silver-haired bat, Canada, 1988 (2)	AF351840
	LC01CA	Hoary bat, Canada, 1992 (2)	AF351845
	ML04CA	Little brown bat, Canada, 1992 (2)	AF351839
Asia 1	INDIA-dog	Dog, India (3)	AY374721
	V029SRL	Dog, Sri Lanka, 1986 (1)	AY854596
Asia 2	Mdn127-48	Unknown sp., Philippines (unpub. data)	AB070798
	Phil23-01	Unknown sp., Philippines (unpub. data)	AB070759
Asia 3	FL01-08IDN	Dog, Indonesia (unpub. data)	AB154216
	SC01-68IDN	Cat, Indonesia (unpub. data)	AB154208
	SW01-11IDN	Dog, Indonesia (unpub. data)	AB154239
Asia 4	8738THA	Human, Thailand 1983 (4)	U22653
	HM88THA	Human, Thailand (5)	AY219002
Arctic Group 1	1422	Arctic fox, Alaska (6)	AY352501
	1578T1ON	Striped skunk, Ontario Canada, 1991 (7)	L20673
	4795	Dog, Alaska (6)	AY352498
	743a	Arctic fox, Yakutia, Russia (6)	AY352488
	RV250	Rodent, Tuva, Russia (6)	AY352480
	V875GLD	Unknown sp., Greenland, 1994, this study	DQ521213
	V886GLD	Fox, Greenland, 2001 (1)	AY854602
	T5ARCCA	Dog, Canada, 1993 (8)	U03769
Arctic-like Group 2	196p	Bovine, Pakistan, (6)	AY352495
	RV61	Human, India, (6)	AY352493
	V028NEP	Dog, Nepal, 1989 (1)	AY854597
	V119NEP	Dog, Nepal, 1989, this study	DQ521214
	V121NEP	Dog, Nepal, 1989 (1)	AY854598
	V704IRN	Sheep, Iran (9)	DQ521212
Arctic-like Group 3	304c	Steppe fox, Chita, Russia (6)	AY352459
	857r	Raccoon dog, Chabarovsk, Russia (6)	AY352458
	Komatsugawa	Dog, Japan (6)	AY352494
	KRH2-04	Raccoon, South Korea, 2004 (10)	AY730595
	SKRDG0203CW	Dog, South Korea, 2002 (11)	DQ076124
	SKRRD0204CW	Raccoon dog, South Korea, 2002 (11)	DQ076125
	SKRRD9902PJ	Raccoon dog, South Korea, 1999 (11)	DQ076121
	SKRRD9903YG	Raccoon dog, South Korea, 1999 (11)	DQ076131
	V737KOR	Raccoon dog, South Korea (1)	AY854601
African canid 2	8801CAM	Dog, Cameroon, 1987 (4)	U22634
	V461NIG	Dog, Nigeria, 1996 (1)	AY854600
African mongoose	1500AFS	Yellow mongoose, South Africa, 1987 (4)	U22628
Cosmopolitan	867WSKCA	Striped skunk, Canada, 1992 (12)	AF344306
	8693GAB	Dog, Gabon, 1986 (4)	U22629
	8706ARS	Red fox, Saudi Arabia, 1987 (4)	U22481
	8708NAM	Kudu, Namibia, 1987 (4)	U22632
	9107MAR	Human, Morocco, 1990 (4)	U22852
	9142EST	Raccoon dog, Estonia, 1985 (4)	U22476
	9147FRA	Red fox, France, 1991 (4)	U22474
	9339EST	Raccoon dog, Estonia (13)	U42707
	9221TAN	Dog, Tanzania, 1992 (4)	U22645
	RV259	Red fox, Kazakhstan (6)	AY352491
	RV260	Red fox, Omsk, Russia (6)	AY352465
	RV299	Red fox, Tula, Russia (6)	AY352479
	RV1596	Red fox, Pskov, Russia (6)	AY352474
	V590MX	Dog, Mexico, 1990 (14)	AY854589
	V685IRN	Goat, Iran, 2000 (9)	AY854580
	V686IRN	Bovine, Iran, 2000 (9)	AY854581
	V1069CU	Goat, Cuba, 2000 (1)	AY854552

	CVS	Laboratory strain	D42112
	PV	Laboratory strain	NC_001542
EBLV-2	9018HOL	Myotis bat, the Netherlands, 1986 (*15*)	U22847

Appendix Table References

1. Nadin-Davis SA, Torres G, de los Angeles Ribas M, Guzman M, Cruz de la Paz R, Morales M, et al. A molecular epidemiological study of rabies in Cuba. Epidemiol Infect. 2006;134:1313–24.
2. Nadin-Davis SA, Huang W, Armstrong J, Casey GA, Bahloul C, Tordo N, et al. Antigenic and genetic divergence of rabies viruses from bat species indigenous to Canada. Virus Res. 2001;74:139–56.
3. Jayakumar R, Tirumurugaan KG, Ganga G, Kumanan K, Mahalinga Nainar A. Characterization of nucleoprotein gene sequence of an Indian isolate of rabies virus. Acta Virol. 2004;48:47–50.
4. Kissi B, Tordo N, Bourhy H. Genetic polymorphism in the rabies virus nucleoprotein gene. Virology. 1995;209:526–37.
5. Hemachudha T, Wacharapluesadee S, Lumlertdaecha B, Orciari LA, Rupprecht CE, La-Ongpant M, et al. Sequence analysis of rabies virus in humans exhibiting encephalitic or paralytic rabies. J Infect Dis. 2003;188:960–6.
6. Kuzmin IV, Botvinkin AD, McElhinney LM, Smith JS, Orciari LA, Hughes GJ, et al. Molecular epidemiology of terrestrial rabies in the former Soviet Union. J Wildl Dis. 2004;40:617–31.
7. Nadin-Davis SA, Casey GA, Wandeler A. Identification of regional variants of the rabies virus within the Canadian province of Ontario. J Gen Virol. 1993;74:829–37.
8. Nadin-Davis SA, Casey GA, Wandeler AI. A molecular epidemiological study of rabies virus in central Ontario and western Quebec. J Gen Virol. 1994;75:2575–83.
9. Nadin-Davis SA, Simani S, Armstrong J, Fayaz A, Wandeler AI. Molecular and antigenic characterization of rabies viruses from Iran identifies variants with distinct epidemiological origins. Epidemiol Infect. 2003;131:777–90.
10. Park YJ, Shin MK, Kwon HM. Genetic characterisation of rabies virus isolates in Korea. Virus Genes. 2005;30:341–7.
11. Hyun BH, Lee KK, Kim IJ, Lee KW, Park HJ, Lee OS, et al. Molecular epidemiology of rabies virus isolates from South Korea. Virus Res. 2005;114:113–25.
12. Nadin-Davis SA, Huang W, Wandeler AI. Polymorphism of rabies viruses within the phosphoprotein and matrix protein genes. Arch Virol. 1997;142:979–92.
13. Bourhy H, Kissi B, Audry L, Smreczak M, Sadkowska-Todys M, Kulonen K, et al. Ecology and evolution of rabies virus in Europe. J Gen Virol. 1999;80:2545–57.
14. Loza-Rubio E, Rojas-Anaya E, Banda-Ruiz VM, Nadin-Davis SA, Cortez-Garcia B. Detection of multiple strains of rabies virus RNA using primers designed to target Mexican vampire bat variants. Epidemiol Infect. 2005;133:927–34.
15. Bourhy H, Kissi B, Tordo N. Molecular diversity of the *Lyssavirus* genus. Virology. 1993;194:70–81.
